# A novel splice site variant in *DEGS1* leads to aberrant splicing and loss of DEGS1 enzyme activity, a VUS resolved

**DOI:** 10.1007/s00439-026-02830-9

**Published:** 2026-05-06

**Authors:** Holly C. Beale, Victor Tse, Joanna Y. Lee, Jon Akutagawa, Yusuph Mavura, Brandon Saint-John, Allison Cheney, Dennis R. Mulligan, Guillermo Chacaltana, Martin Gutierrez, Jessica Tenney, Joseph T. Shieh, Pierre-Marie Martin, Tiffany Yip, Ugur Hodoglugil, Alex J. Fay, Angela N. Brooks, Jessica Van Ziffle, Michael D. Stone, Neil Risch, Jeremy R. Sanford, Patrick Devine, Julie D. Saba, Olena M. Vaske, Anne Slavotinek

**Affiliations:** 1 Department of Molecular, Cell and Developmental Biology, University of California Santa Cruz, Santa Cruz, CA 95064 USA; 2 Genomics Institute, University of California Santa Cruz, Santa Cruz, CA 95060 USA; 3 Center for Molecular Biology of RNA, University of California Santa Cruz, Santa Cruz, CA 95064 USA; 4 Department of Pharmacology, University of Texas Southwestern Medical Center, Dallas, TX 75390 USA; 5 Department of Pediatrics, University of California San Francisco, San Francisco, CA 94143 USA; 6 Division of Hematology, University of California San Francisco, San Francisco, CA 94143 USA; 7 Department of Biomolecular Engineering, University of California Santa Cruz, Santa Cruz, CA 95064 USA; 8 Institute for Human Genetics, University of California San Francisco, San Francisco, CA 94143 USA; 9 Department of Epidemiology and Biostatistics, University of California San Francisco, San Francisco, CA 94158 USA; 10 Biological Systems and Engineering Division, Lawrence Berkeley National Laboratory, Berkeley, CA 94720 USA; 11 Department of Chemistry and Biochemistry, University of California Santa Cruz, Santa Cruz, CA 95064 USA; 12 Division of Medical Genetics, University of California San Francisco, San Francisco, CA 94158 USA; 13 Genomic Medicine Laboratory, University of California San Francisco, San Francisco, CA 94158 USA; 14 Department of Neurology, University of California San Francisco, San Francisco, CA 94143 USA; 15 Division of Child Neurology, University of California San Francisco Benioff Children’s Hospital, San Francisco, CA 94158 USA; 16 Department of Pathology, University of California San Francisco, San Francisco, CA 94143 USA; 17 Genetics and Genomics Services, University of California San Francisco, San Francisco, CA 94143 USA

## Abstract

Pathogenic *DEGS1* variants have been reported in individuals with autosomal recessive hypomyelinating leukodystrophy 18 (HLD18; MIM# 618404). Here we describe three participants with HLD features and a previously unreported homozygous *DEGS1* 5′ splice site variant, c.825+4_825 + 5delAGinsTT (NM_003676.4). We used next-generation DNA and transcriptome sequencing, cell-based splicing assays, and tandem mass spectrometry to detect and characterize the variant’s impact on *DEGS1* expression. We then performed RNA structure probing and conventional antisense oligonucleotide screening to investigate molecular mechanisms for potential therapeutic intervention. We show that the splice site variant: (1) was sufficient to induce exon two skipping in most detected transcripts; (2) resulted in structural changes to the 5′ and 3′ splice site regions using RNA structure probing; and (3) corresponds to plasma sphingolipid profiles consistent with loss of sphingolipid delta(4)-desaturase activity. Our RNA and lipidomic evidence proved that the *DEGS1* variant c.825+4_825 + 5delAGinsTT is pathogenic and suggested a mechanistic model that explains how exon two skipping is induced.

**Supplementary Information:**

The online version contains supplementary material available at 10.1007/s00439-026-02830-9.

## Introduction

Hypomyelinating leukodystrophies (HLD) are a heterogeneous group of heritable, progressive disorders in which the formation of myelin sheaths is disrupted, resulting in neurodegenerative white matter disease that can affect the function of neurons and impair cognitive and motor skills (Parikh et al. 2015; Köhler et al. 2018; Metovic et al. 2024). Clinical manifestations of HLD are highly variable, even for individuals within the same family, but often include motor regression and neurological findings such as dystonia, spasticity, abnormal tone, developmental delay, seizure, nystagmus, cognition and learning impairment (Pant et al. 2019; Mahdieh et al. 2021; Metovic et al. 2024). Currently, twenty-seven genes are associated with HLD in the Online Mendelian Inheritance in Man database (MIM# 312080).

Loss of function variants in *Delta 4-Desaturase*,* Sphingolipid 1* (*DEGS1*) are associated with autosomal recessive hypomyelinating leukodystrophy 18 (HLD18; MIM# 618404). Approximately thirty individuals with HLD18 have been reported in the literature to date (Karsai et al. 2019; Pant et al. 2019; Dolgin et al. 2019; Yan et al. 2021; Wong et al. 2023; Hülsmeier et al. 2023; Afridi et al. 2024). *DEGS1* is a three exon gene that functions in the *de novo* ceramide biosynthesis pathway. The product of the *DEGS1* gene, DES1, is an enzyme that catalyzes the conversion of dihydroceramide (DHCer) to ceramide (Cer) through the introduction of a double bond into the backbone of the sphingolipid (Ternes et al. 2002). The conversion of saturated to unsaturated sphingolipids is an essential process that maintains the myelin that surrounds and protects the axons of neurons (Schmitt et al. 2015).

Aberrant splicing of precursor messenger RNA (pre-mRNA) is a common defect in many inherited diseases (Wang and Aifantis 2020; Lord and Baralle 2021). These gene expression errors typically arise from variants at the consensus splice site sequences that define exon-intron boundaries. Accurate expression of the *DEGS1* gene requires removal of two introns from the pre-mRNA. To our knowledge, no publications report splice site variants in HLD18 patients. However, the ClinVar database contains three *DEGS1* splice site variants: Variation ID #2,502,429 is reported as likely pathogenic in an affected individual; #1,514,221 is reported as likely pathogenic in an individual with no reported phenotype; and #1,481,460 is of uncertain significance found in an individual with no reported phenotype (Supplemental Table 1). These results suggest a potential role for aberrant splicing in HLD.

In this study, we report the discovery and characterization of a *DEGS1* splice site variant found in three participants with clinical features consistent with HLD18 from two unrelated families. We then performed RNA structure probing and conventional antisense oligonucleotide (ASOs) screening “walks” to investigate molecular mechanisms for possible therapeutic intervention. Our results elucidate a mechanistic understanding of this *DEGS1* pathogenic variant, providing a foundation for future strategies seeking to develop splice-modulating therapeutics for HLD patients.

## Materials and methods

Written, informed consent was obtained from the parents of participants one, two and three using a consent form from the Program in Prenatal and Pediatric Genomic Sequencing (P^3^EGS) study, part of the National Human Genome Research Institute (NHRGI) Clinical Sequencing Evidence Generating (CSER) consortium, that was approved by the Institutional Review Board (IRB) at the University of California, San Francisco (UCSF).

### Exome sequencing and variant identification

Exome sequencing was performed as previously described on DNA isolated from blood or saliva submitted by participants and family members, respectively (Slavotinek et al. 2023). Briefly, targeted regions were selected with the xGen Whole Exome Panel kit v1 (Integrated DNA Technologies) and sequenced using the Illumina HiSeq 2500 sequencing system with v3 chemistry generating 100 bp paired-end reads in rapid run mode. Mean depths of coverage for participants one and two were 94X reads and 102X reads, and 99.5% and 99.6% of exons, respectively, had at least 10X coverage. The resulting DNA sequences were mapped to the human genome GRCh37/hg19.

Sequence variant detection, filtering, ranking and annotation was performed using Opal Clinical (Fabric Genomics) and Moon Diploid. The variants found in the participants were compared to variants in other family members to annotate the variants based on their inheritance patterns: *de novo*, homozygous, compound heterozygous and inherited heterozygous. The inherited heterozygous variants were filtered based on the following key words and Human Phenotype Ontology (HPO) terms. Key word(s) and/or HPO terms used to filter and rank variants in the first participant included: severe global developmental delay (HP:0011344), contractures (HP:0001371), seizures (HP:0001250), generalized hypotonia (HP:0001290), cortical visual impairment (HP:0100704), optic atrophy (HP:0000648). Key word(s) and/or HPO terms used to filter and rank variants in the second participant included: seizures (HP:0001250), global developmental delay (HP:0001263), hypertonia (HP:0001276), microcephaly (HP:0000252), spastic quadriparesis (HP:0001285), cerebral palsy (HP:0100021), facial dysmorphism (HP:0001999), hirsutism (HP:0001007), cerebellar atrophy (HP:0001272), white matter abnormalities (HP:0002500), hypomyelination (HP:0003429). The variant data were also analyzed using a manual pipeline whereby bioinformatics experts from UCSF Genomic Medicine Laboratory manually curated the inherited heterozygous variants with the gene lists and keywords in Supplemental Table 2. In the third participant, who was the sibling to the second patient, DNA from whole blood was analyzed using a Leukodystrophy and Genetic Leukoencephalopathy Panel (Invitae, Inc.).

We identified a splice site variant in *DEGS1* which replaced the nucleotides AG with TT at position 4 of the exon/intron boundary (NM_003676.4) c.825+4_825 + 5delAGinsTT in the exome sequencing. This variant was confirmed to be homozygous in participants one and two by Sanger sequencing. Briefly, amplification of genomic DNA with forward primer 5′- ACCGATTTTGAGGGCTGGTT − 3′ and reverse primer 5′- ATGAACTGCTTGGACTGACA − 3′, was followed by sequencing with a BigDye Terminator 3.1 Cycle Sequencing Kit and Genetic Analyzer 3500 (ThermoFisher Scientific).

### Genetic relatedness estimation using PC-relate

We estimated the relatedness of participants and parents from two families using the program PC-Relate (Conomos et al. 2016) as previously described using six principal components (Mavura et al. 2024).

### Transcriptome sequencing and isoform analysis

RNA was extracted from whole blood (Maxwell^®^ RSC simplyRNA Blood Kit, Promega catalog # AS1380) from participant one and an unrelated control without any pathogenic variants or a variant of uncertain significance (VUS) in *DEGS1*. Libraries were prepared with the.

KAPA Stranded mRNA-Seq Kit with KAPA mRNA Capture Beads (Roche catalog #07962193001). 150-base paired end sequence was generated from libraries on an Illumina Novaseq 6000 sequencer using an S4 flow cell. Participant one data had 82.6 million total pairs of reads, of which 45.1 million were Mapped Exonic Non-Duplicate (MEND) reads (Beale et al. 2021). The control data had 87.7 and 22.9 million total and MEND reads, respectively. For comparison to reference genome and transcripts, reads were aligned by STAR v2.4.2a using indices generated from the human reference genome GRCh38 and the human gene models GENCODE 23. To assemble novel transcripts, hisat2:2.2.1 was used to align reads to GRCh38 (with the index grch38_snp_tran.tar.gz downloaded from ftp://ftp.ccb.jhu.edu/pub/infphilo/hisat2/data/; current download links can be found at https://daehwankimlab.github.io/hisat2/download/). Novel and reference transcripts were identified using Stringtie 2.1.6, with and without Gencode v38 as a guide. Additionally, Stringtie was run on aligned reads after duplicates were removed with samblaster 0.1.26. All outputs were merged to generate a gene transfer format (GTF) file with consistent identifiers containing all reference and de novo transcripts, and transcript quantification was repeated with Stringtie using the merged GTF as a guide. The fraction of *DEGS1* expression accounted for by each isoform was calculated based on the TPM of each isoform. We report isoform abundance using duplicate-free data; the abundances based on data inclusive of duplicate reads are similar. A sashimi plot was generated with IGV (Robinson et al. 2011).

### *DEGS1* junction usage analysis

Percent-spliced (PS) values for splice junctions were computed with mesa v1.0.0 (https://github.com/BrooksLabUCSC/mesa) for participant one, the RNA-Seq control, and 670 whole blood samples from the Genotype-Tissue Expression (GTEx) v8 dataset (GTEx Consortium 2017). Reads were aligned to the hg38 reference genome using STAR 2.4.2a. A PS value for a splice junction (inclusion) is calculated by the total read count for that junction, divided by the total of the inclusion and all exclusion junctions. The exclusion are reads containing any other splice junction that overlaps the inclusion splice interval that is being quantified. These intervals are considered mutually exclusive, and represent some form of alternative splicing. A distribution of PS values from the GTEx whole blood samples was used to calculate the quartile ranges. Outlier splicing events were defined as events that were larger than the third quartile (Q3) + 1.5 * the interquartile range (IQR) or smaller than the first quartile (Q1) − 1.5 * IQR. PS values from participant one and the RNA-Seq control were then compared to these cutoffs.

### *DEGS1* splicing reporters

Reference and variant *DEGS1* exon two splicing reporters were generated and validated as previously described (Tse et al. 2024). Briefly, we used the same site-directed mutagenesis and primer assembly strategy to generate inserts corresponding to the reference or pathogenic variant context of *DEGS1* exon two and flanking introns. The primers used to generate the reference and variant inserts can be found in Supplemental Table 3. Using homology-based cloning technology (In-Fusion HD Cloning kit, Takara Bio), the inserts are then cloned into the pACT7_SC14 heterologous *HBB* splicing reporter (Rothrock et al. 2003). Sanger sequencing was then employed to confirm the successful cloning and identity of each splicing reporter (UC Berkeley, DNA Sequencing Center).

### Cell-based in vivo splicing reporter assays

HEK293T cells (ATCC) were cultured in 6-well tissue culture plates (CytoOne, USA Scientific) using Dulbecco’s Modified Eagle Medium (Gibco™, supplemented with 10% fetal bovine serum (FBS)) at 37 °C with a CO_2_ level of 5%. Prior to the time of performing the assays, cells were grown to a confluency of ~ 60–80%. 2.5 µg of each splicing reporter was transiently transfected into HEK293T cells using Lipofectamine 2000 (ThermoFisher). At 24 hours post transfection, cells were harvested and prepared for total RNA purification using the Direct-zol RNA Miniprep kits from Zymo Research.

### Antisense oligonucleotides (ASOs)

*DEGS1* exon two ASOs were designed by taking the reverse complement of the coding sequence, specifying sequences of *k*-mer length, which were then annotated with desired modifications to the ribose sugar. To infer nuclease resistance and in vivo stability to ASOs, the 2’OH contained a methoxyethyl modification (2’MOE) and the phosphate backbone was modified to a phosphorothioate backbone. *DEGS1* ASOs were designed to be 18 nucleotides in length and were synthesized by Integrated DNA Technologies (IDT). The sequences of the ASOs can be found in Supplemental Table 4.

### *DEGS1* reference and variant ASO walks

HEK293T cells (ATCC) were cultured in 96-well tissue culture plates (Perkin Elmer) using Dulbecco’s Modified Eagle Medium (Gibco, supplemented with 10% FBS) at 37 °C with a CO_2_ level of 5%. Prior to performing the assays, cells were grown to a cell confluency of ~ 60–80%. 250 ng of reference or variant *DEGS1* splicing reporters were transiently co-transfected with 10 pmol of each ASO into HEK293T cells using Lipofectamine 2000 (ThermoFisher). After 24-hours post transfection, cells were then harvested and prepared for total RNA purification using the *Quick*-DNA/RNA Viral MagBead kit from Zymo Research, in which this workflow has been automated on the Agilent Bravo.

### 2-step RT-qPCR and qualitative analysis of splicing reporter assays

First-strand cDNA synthesis of total RNA using Multiscribe Reverse Transcriptase (Applied Biosystems) and 5’FAM end-labeled PCR amplification of mRNA reporter isoforms was performed following protocols as described (Tse et al. 2024). The resulting amplicons corresponding to reference or pathogenic variant mRNA were then analyzed using gel electrophoresis to empirically evaluate mRNA isoforms detected.

### Quantitative analysis of splicing reporter assays using fragment analysis

The abundance of each 5’FAM end-labeled amplicon was quantified and analyzed following protocols as described (Tse et al. 2024).

### Calculating splicing efficiency using percent-spliced-in (PSI) index formula

Quantification of reference or variant *DEGS1* exon two splicing efficiency was determined following protocols as described (Tse et al. 2024).

### *DEGS1* reference and variant exon two RNA synthesis

*DEGS1* exon 2 RNA(s) were synthesized by T7 RNA polymerase using a *DEGS1* reference/variant construct containing intron spanning regions as a template. RNA was purified using standard agarose gel extraction followed by overnight ethanol precipitation.

### In-vitro mutational profiling coupled with high throughput sequencing (MaP-seq)

MaP-seq experiments were performed and data were analyzed with RNAFramework and RNAstructure as previously described (Tse et al. 2024).

### Human subjects for healthy control blood collection

Pediatric plasma samples from nine healthy children and young adults were obtained from the Benioff Children’s Hospital Oakland clinical laboratory in a deidentified state after selection by the clinical laboratory manager. Blood was collected from participants in accordance with an approved IRB protocol at UCSF. Inclusion criteria were male and female pediatric subjects 0–20 years of age who were undergoing preoperative lab testing prior to elective surgery. Exclusion criteria were any subjects with a metabolic, malignant, infectious, autoimmune or hemolytic diagnosis. Samples were maintained at 4 °C and processed within 24 h of the time of collection.

### Plasma isolation

Samples of EDTA chelated whole venous blood were processed by centrifugation at 4 °C at 350 x g for five minutes. Supernatant was transferred to a new tube and centrifuged at 8,050 x g at 4 °C for five minutes. The clear plasma was transferred to new microcentrifuge tubes, snap frozen with dry ice, and stored at -80 °C until lipid extraction. Hemolyzed blood samples were excluded from the analysis.

### Liquid chromatography mass spectrometry (LC-MS/MS) detection of sphingolipids

Plasma sphingolipids were extracted as previously described (Bielawski et al. 2006). Detection of sphingolipid and data processing methods were used as previously described (Suh et al. 2018). Data processing was performed using the Agilent quantitative analysis application. All samples were analyzed using triplicates. All data are expressed as the mean ± standard deviation (SD). Differences were examined for significance using the two-tailed Student’s test (t test), with *p* < 0.05 as the cutoff for statistical significance.

## Results

### Clinical presentation

**Fig. 1:**
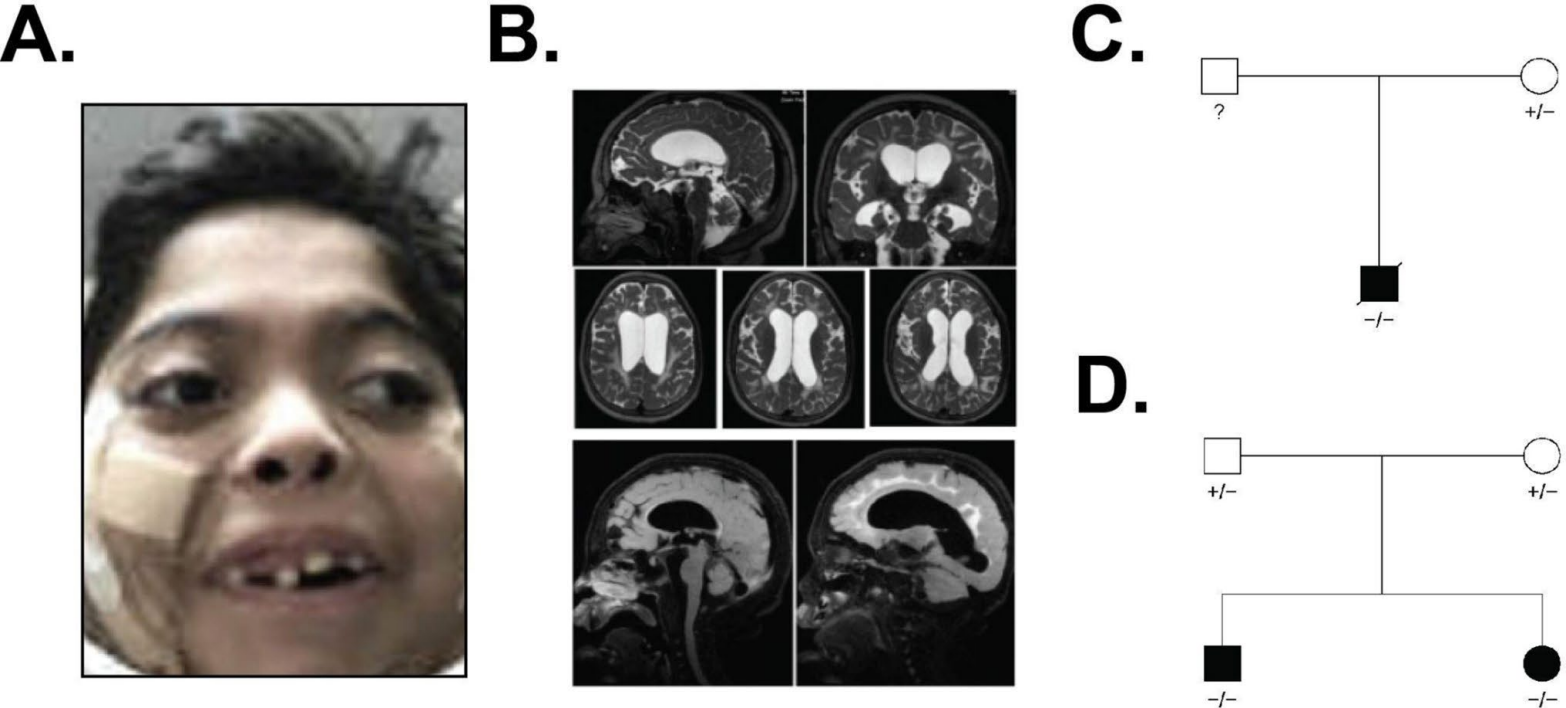
Clinical presentation of unrelated patients with a previously unreported homozygous, 5′ splice site variant in *DEGS1*. **A** Facial photograph of the first participant, showing prominent eyes with partial ptosis, a depressed nasal bridge, anteverted nares, broad and wide mouth and micrognathia. He has thick eyebrows and long eyelashes. **B** Magnetic resonance imaging (MRI) of the brain for participant one showed profound paucity of the white matter, thinning of the corpus callosum, volume loss of the midbrain and cerebellar vermis, and periventricular heterotopia of the left frontal horn. **C** Pedigree of family one and participant one. **D** Pedigree of family two with participant two (male) and participant three (female)

We present three participants from two families (Table 1). All participants had severe motor delays, with failure to achieve independent sitting by 15 months of age (participant three), four years (participant one), and 14 years (participant two). All three participants were noted to have nystagmus in the neonatal period. All had feeding difficulties with resultant failure to thrive, microcephaly, abnormal tone, and weakness. None were able to babble or acquire single words. Participants one and two manifested seizures and developed limb contractures involving the wrists and knees and, in one, the elbows. Participant one (Fig. 1A) developed respiratory failure and was deceased at seven years eight months, whereas participant two suffered respiratory decline at ten years of age and required a tracheostomy at twelve years. In participant one, magnetic resonance imaging (MRI) of the brain showed abnormal myelination development (Fig. 1B). For a full description of clinical findings of these participants see Supplemental Table 5.

**Table 1:** A comparison of clinical features between published reports of patients with biallelic deleterious variants in *DEGS1*, and the patients in this report

	Reported patients (see Supplemental Table 5)	Patients in this report
Demographic		
Gender	16 Male, 14 Female	2 Male, 1 Female
Ancestry	Panethnic	Central American
Postnatal growth		
Acquired microcephaly	8/24	3/3
Acquired failure to thrive	15/21	3/3
Feeding difficulties	9/25	3/3
Development		
Motor acquisition - sitting	12/23	0/2
Motor acquisition - standing	5/22	0/2
Speech acquisition – single words	5/24	0/2
Speech acquisition – sentences	5/21	0/2
Neurological		
Nystagmus	16/23	1/1
Increased tone/spasticity	25/26	2/3
Axial/limb dystonia	12/21	2/3
Seizures	19/26	1/2

### Detection of a splice site variant of uncertain significance in *DEGS1*

**Fig. 2:**
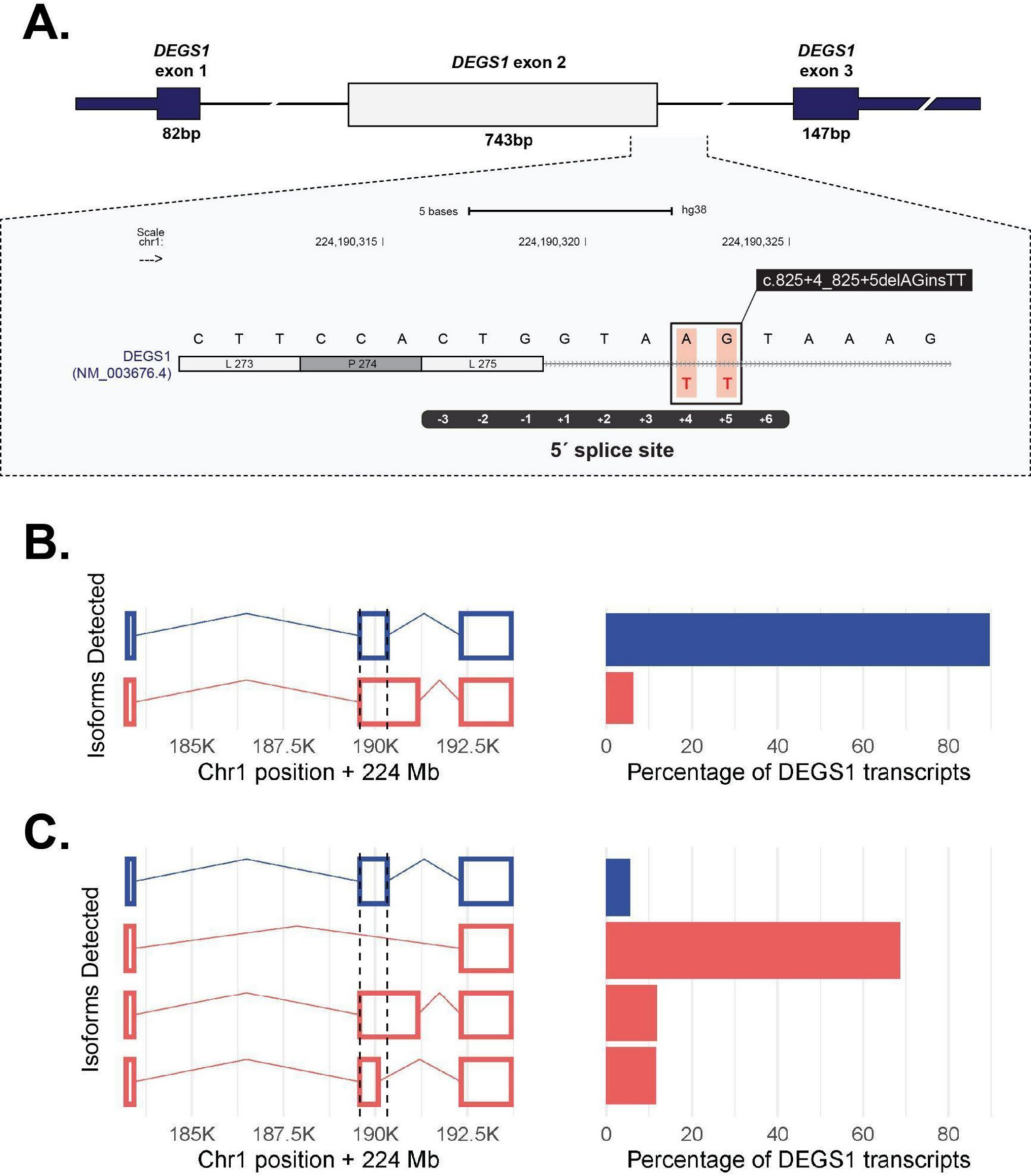
*DEGS1* variant and isoforms detected. **A** The c.825+4_825 + 5delAGinsTT variant maps to the 5′ splice site of exon 2. The position of the variant is boxed in the enlarged inset. **B** *DEGS1* isoforms (left) present at more than 5% in the control and the corresponding abundance (right). The isoform plots depict canonical (blue) and non-canonical isoforms (red). The canonical transcript comprised most of the transcript molecules. **C** *DEGS1* isoforms and abundance in participant one. Transcript assembly identified two non-canonical transcripts not detected in the control. The novel transcript that skips exon 2 is the predominant transcript

All participants shared a homozygous *DEGS1* (NM_003676.4) 5’ splice site variant (Fig. 1C, D), NM_003676.4:c.825+4_825 + 5delAGinsTT, which replaced the nucleotides AG with TT at position 4 and 5 of the exon/intron boundary (Fig. 2A; NC_000001.10:g.224378025_224378026delinsTT (GRCh37)). In addition, the variant was heterozygous in all available parents (Supplemental Fig. 1A, B).

This variant was annotated as a variant of uncertain significance (VUS) because there was insufficient evidence from prior reports or functional studies to infer pathogenicity. This classification was based on the American College of Medical Genetics and Genomics (ACMG) guidelines (Richards et al. 2015). Specifically, the variant was classified as a VUS as it was: (1) absent from population databases (e.g., gnomAD, dbSNP, TOPMed, DiscovEHR) (Sherry et al. 2001; Dewey et al. 2016; Taliun et al. 2021; Chen et al. 2024) and (2) has not previously been published in the literature or associated with disease in databases such as ClinVar. Both families self-reported their ancestry as Guatemalan, suggesting a potential founder mutation in that ancestry (Ziyatdinov et al. 2023). We also determined that the two families are only distantly related, at least to the fourth degree (Supplemental Fig. 2). However, we also noted that the splice site variant was carried on a shared haplotype extending thousands of nucleotides between the three patients and two families, confirming a common founder origin.

### Exon 2 of *DEGS1* is skipped in transcripts from participant one

Two splice effect prediction algorithms indicated the variant would have a deleterious effect on splice sites. SpliceAI (Jaganathan et al. 2019) reported a 94% probability of donor loss (https://spliceailookup.broadinstitute.org, accessed 3/4/2025), and MaxEntScan (Yeo and Burge 2004) reported reduced strength score for the 5′ splice site from 10.65 for the reference sequence to 3.5 for the variant sequence (Supplemental Table 6).

To determine whether the splice site variant had a functional impact on *DEGS1* splicing, we compared transcripts in whole blood from an individual without pathogenic or uncertain variants in *DEGS1* (the control), participant one, and GTEx, which is a large cohort of normal samples. We first quantified *DEGS1* isoforms. In whole blood in GTEx, the MANE select transcript ENST00000323699 (NM_003676) comprised 97.6% of expressed reference transcripts (GTEx transcript browser https://www.gtexportal.org/home/transcript, page accessed 02/11/2025). When we assembled *DEGS1* transcripts from RNA sequencing data, 90% of *DEGS1* transcripts detected in the control sample were the reference transcript NM_003676 (Fig. 2B; Supplemental Fig. 3). In contrast, NM_003676 comprised only 6% of the *DEGS1* transcripts in participant one. Instead, the most abundant *DEGS1* transcript detected in participant one (69%) corresponded to a novel transcript that differed from NM_003676 only by skipping the second of three exons (Fig. 2C; Supplemental Tables 7 and 8). This novel isoform is predicted to correspond to the amino acid change NP_003667.1:Ala28Glyfs*7. We also observed a transcript relying on a cryptic splice site at NC_000001.10:g.224,377,775 (GRCh37). It comprised 12% of *DEGS1* transcripts in participant one, and is predicted to correspond to the amino acid change NP_003667.1:Thr195_Val276_del. The deletion includes sequences that code for transmembrane domains.

We next quantified splice junction usage among the three NM_003676 exons. As one would expect from the prevalence of the three-exon NM_003676 in normal blood, evidence for usage of exon 1-2 junctions and exon 2-3 junctions was abundant in the control and GTEx whole blood data. In participant one data, usage of those exons fell below the down-outlier threshold (Supplemental Fig. 4). The exon 1-3 junction, which is not present in reference gene models, was abundant in participant one and was not found in the control. In GTEx whole blood data, only 44% of samples used the *DEGS1* exon 1-3 junction at all, and at most, that junction usage comprised 10% of *DEGS1* junction usage. In comparison, it comprised 56% of junction usage in participant one (Supplemental Table 9).

### The splice site variant is sufficient to induce complete skipping of *DEGS1* exon 2 

**Fig. 3:**
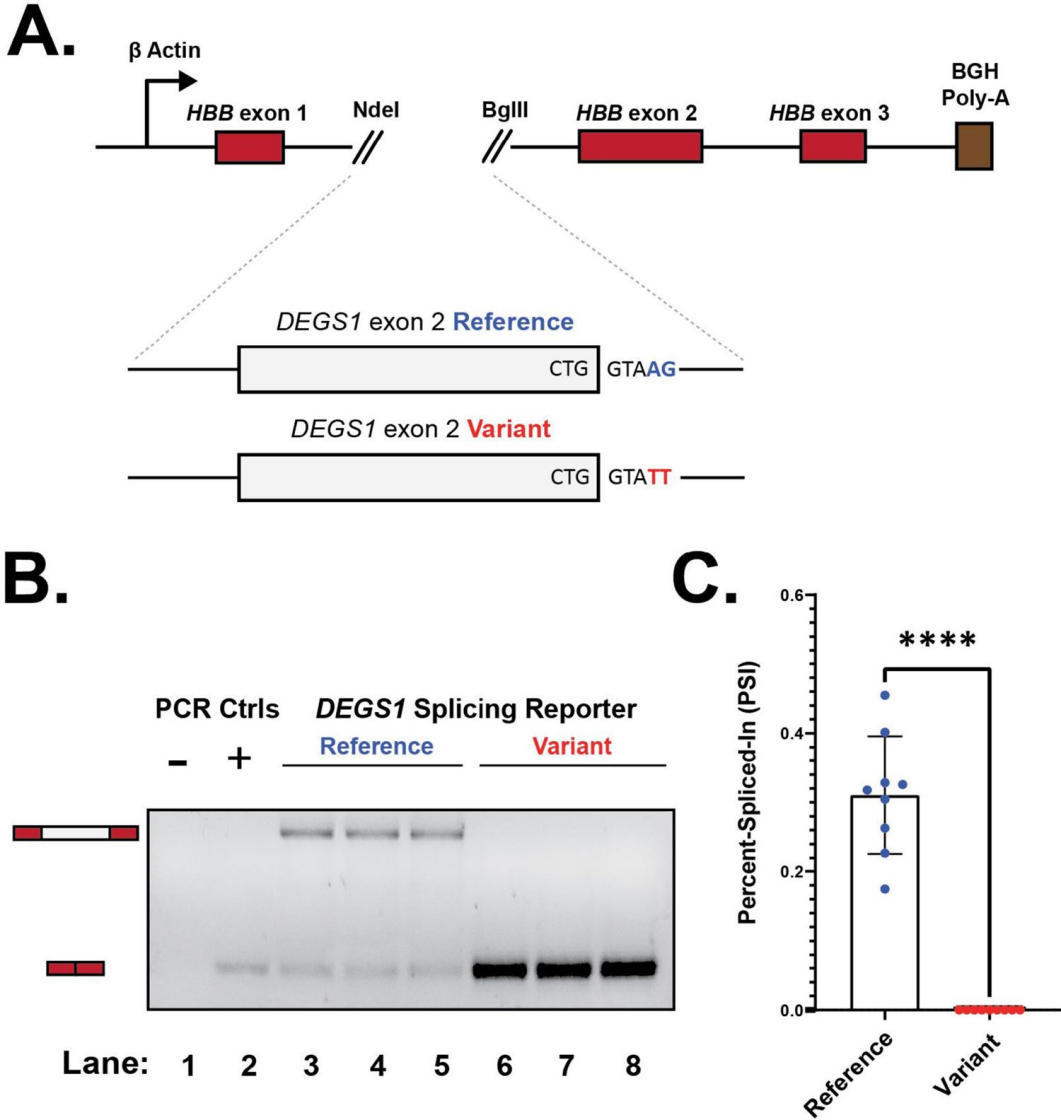
Splice site variant is sufficient to induce skipping of *DEGS1* exon 2. **A** Schematic depicting the heterologous splicing reporter system used to assay the functional impact the novel splice site variant has on *DEGS1* exon 2 splicing, relative to the reference. **B** A representative agarose gel showing the variant’s effect on *DEGS1* exon 2 splicing. As shown in the annotation above the representative agarose gel, controls include a no template reaction (lane 1) and a positive control for exon skipping (lane 2). Lanes corresponding to the splicing reporters that assayed the splice site variant or reference sequence context of *DEGS1* exon 2 are indicated respectively. Expected mRNA isoforms including or excluding the *DEGS1* exon 2 are also annotated to the left of the agarose gel. **C** Percent-spliced-in (PSI) plot quantifying the results shown in **B**, measuring the splice site variant’s impact on *DEGS1* exon 2 inclusion. PSI refers to the fraction of mRNA reporter isoforms that include the exon of interest, relative to the total population of mRNA reporter isoforms. Statistical significance between comparisons shown is denoted by asterisks (i.e., ****) that represent *P* ≤ 0.0001. Statistical significance was determined using analysis of variance (ANOVA), and Dunett’s post-hoc test. Each condition tested and presented contains nine independent/biological replicates

To experimentally validate computational predictions and our RNA-seq data, we performed cell-based splicing reporter assays to examine *DEGS1* exon 2 inclusion in the reference and variant contexts (Fig. 3A). Relative to the reference (lanes 3–5), the splice site variant induced complete and significant skipping of *DEGS1* exon 2 from the splicing reporter (lanes 6–8) (Fig. 3B, C). This finding recapitulated and supported the predominant exon 2 skipping isoform detected from RNA-seq of participant one (Fig. 2C). Together, these findings show that this novel splice site variant was sufficient by itself to cause significant skipping of *DEGS1* exon 2. .

### Splice site variant drives refolding of RNA secondary structures that may occlude splicing regulatory sequence

**Fig. 4:**
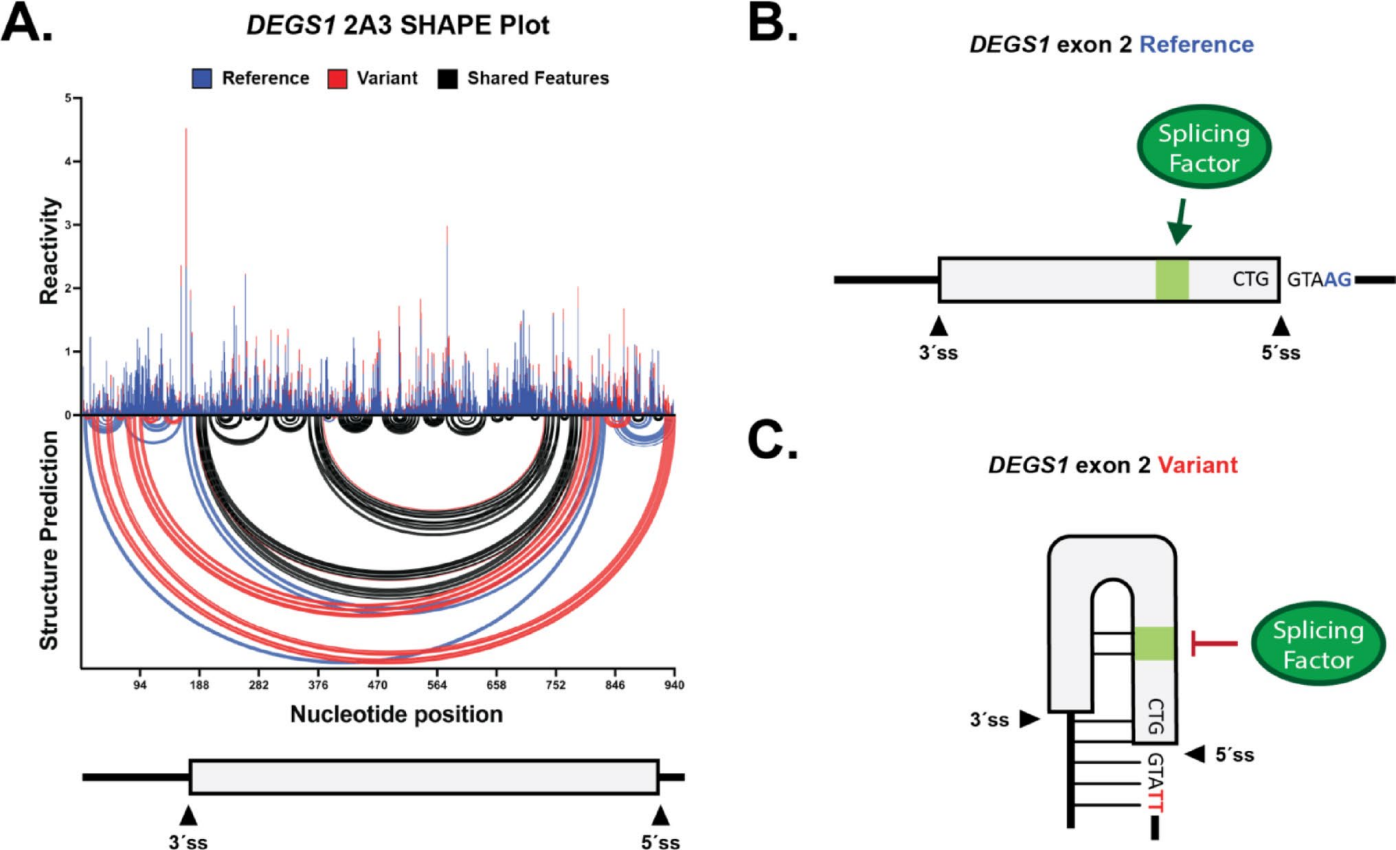
Splice site variant drives intramolecular refolding of RNA secondary structures of *DEGS1* exon 2. **A** A normalized SHAPE reactivity versus structure prediction plot comparing the RNA folding profiles for the reference and variant sequence context of *DEGS1* exon 2 (depicted in blue and red arcs, respectively). Black arcs represent RNA folding features that are found in both the reference and variant context. The top part of the plot shows the normalized SHAPE reactivity for each nucleotide. The bottom part of the plot shows SHAPE-constrained structure predictions represented by intramolecular base pairing interactions; secondary structure formation is denoted by arcs joining different regions of the RNA sequence context. A schematic model of *DEGS1* exon 2 and its flanking introns is also shown at the bottom of the plot to illustrate relative positions of RNA structure data. All SHAPE data analysis was performed in RNA Framework. **B**,** C** Simplified models of *DEGS1* exon 2 in the reference and splice site variant context. Position of splice sites are indicated. Promoted or inhibited interactions between splicing factor and splicing regulatory sequences are depicted. Base pairing is indicated by horizontal solid lines between regions, as seen in (**C**)

To understand the mechanistic impacts of the variant on splice site selection, we characterized the RNA structure and splicing regulatory sequence landscapes. Since RNA structure formation has been implicated to have clinical relevance and can lead to improper splicing (Waldern et al. 2022), we investigated the potential for *DEGS1* exon 2, an unusually large exon, to form local and long-range RNA secondary structures. To test the hypothesis that this splice site variant can affect the RNA folding landscape of *DEGS1* exon 2, we used SHAPE-MaP-seq (*s*elective 2’-*h*ydroxyl *a*cylation analyzed by *p*rimer *e*xtension and *m*ut*a*tional *p*rofiling coupled to high-throughput sequencing) to chemically probe the accessibility of the reference and variant pre-mRNA context corresponding to *DEGS1* exon 2. SHAPE-MaP-seq revealed striking differences between the RNA structure profiles of the reference and variant (Fig. 4A, compare blue to red; Supplemental Fig. 5; Supplemental Fig. 6). In addition to further weakening of the 5′ splice site by the variant through its sequestration in a long-range structure, we also saw that the 3′ splice site in the variant context is now less accessible due to refolding (Supplemental Fig. 7). The data suggest the pathogenic variant drives long-range refolding of RNA structures that weaken the accessibility of the 5′ and 3′ splice sites.

The defective splice site in *DEGS1* exon 2 is intriguing because the c.825+4_825 + 5delAGinsTT variant leaves the conserved + 1 and + 2 positions of the consensus 5’ splice site sequence intact (Wong et al. 2018). A similar splice site variant in exon 20 from the *IKBKAP* gene also causes aberrant splicing, which can be corrected by splice-modulating ASOs targeting splicing silencer elements (Sinha et al. 2018). Additionally, we previously discovered that destabilizing RNA structures with ASOs can enhance the splicing of *F8* exon 16 (Tse et al. 2024). We hypothesized that applying the same discovery approaches from *IKBKAP* and *F8* might benefit *DEGS1* exon 2 to identify regulatory sequences that splice-modulating ASOs can target to enhance inclusion of the pathogenic variant.

A conventional ASO walk did not identify any splice-modulating ASOs for the pathogenic variant, but the same walk on the reference context revealed striking splicing inhibition by multiple ASOs (Supplemental Fig. 8). The sequence of successful inhibitory ASOs overlapped with putative binding sites for serine and arginine-rich splicing factors known to enhance splicing, as indicated by our RBPmap analysis (Supplemental Fig. 9) (Paz et al. 2014). Sequences identified to inhibit or enhance splicing are indicated by red or green in Supplemental Fig. 8D, respectively. These corresponding ASO sequences are found in Supplemental Table 4. The ASO walk results from the reference, coupled to an appropriate non-targeting ASO control, demonstrate that on-target effects by ASOs designed to target *DEGS1* exon 2 and flanking introns in this study have been achieved. The inability to enhance splicing of the pathogenic variant is likely due to significant, weakened base pairing between the + 4 and + 5 positions of the 5’ splice site and U1 snRNP (Wong et al. 2018).

Our data also suggested an additional deleterious mechanism where this splice site variant rearranged the splicing regulatory landscape of *DEGS1* exon 2 (Fig. 4B, C). The reference walk defined sequence elements that control exon two splicing. Combining this finding with our RBPmap analysis and RNA structure data indicates this variant may weaken exon 2 accessibility between splicing factors and functional regulatory sequences. These identified changes to RNA folding dynamics, and the discovery of splicing regulatory sequences, may be useful for future strategies developing therapeutic agents to modulate the splicing of *DEGS1* exon 2 in a subset of HLD patients.

### Reduced desaturase activity observed in the tested probands

**Fig. 5:**
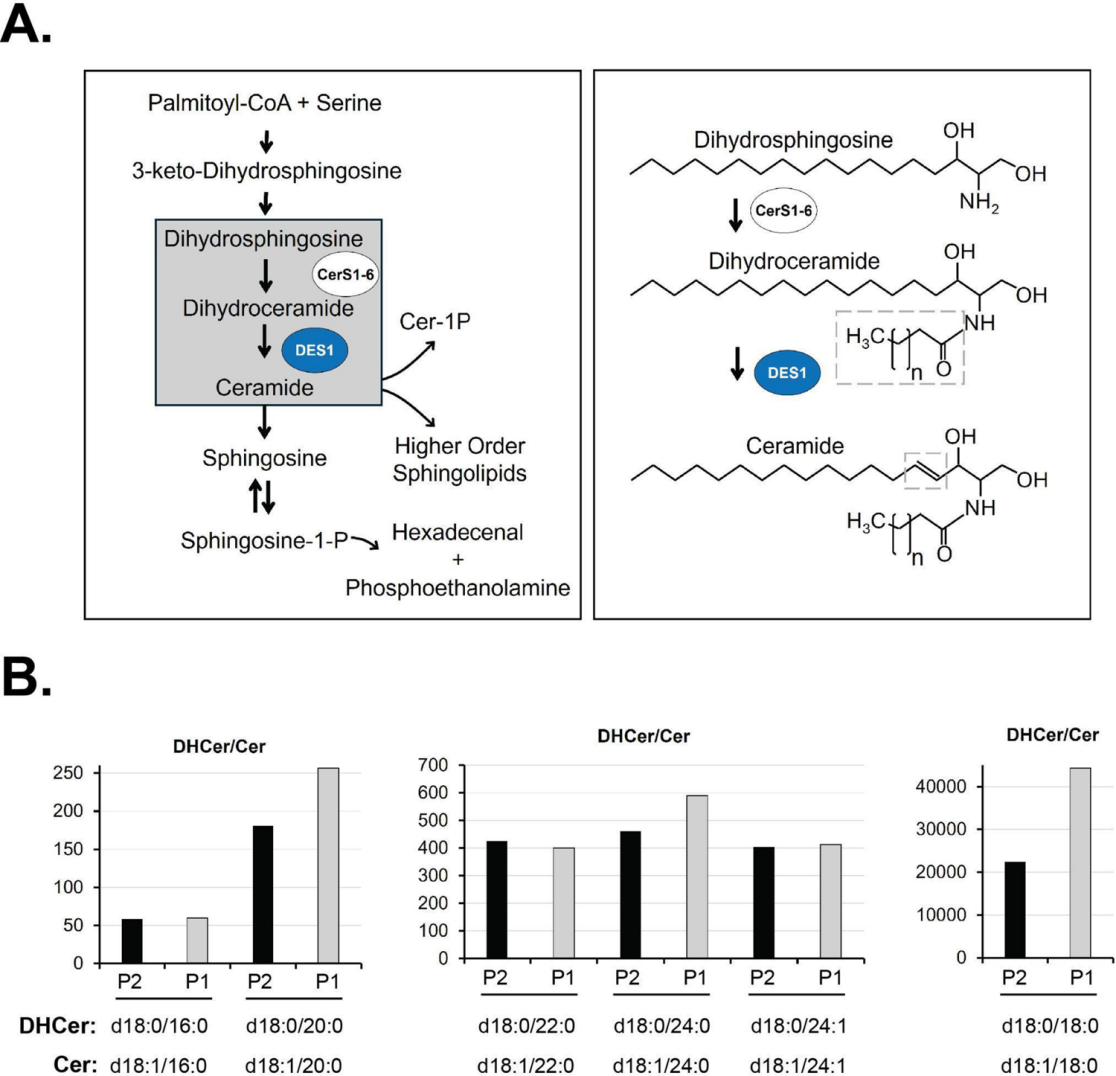
Splice site variant leads to loss-of-function. **A** The role of DES1, the product of the *DEGS1* gene, in sphingolipid metabolism. LEFT: Sphingolipid biosynthesis begins with condensation of serine and palmitoylCoA, resulting in 3-keto dihydrosphingosine. This is reduced, forming dihydrosphingosine. Ceramide synthases (CerS) acylate dihydrosphingosine at the free amino group with fatty acids of varying chain lengths and saturation. DES1 desaturates the dihydrosphingosine backbone, introducing a double bond that converts dihydrosphingosine to sphingosine, and correspondingly converts dihydroceramide into ceramide. Formation of all ceramides requires this step. RIGHT: The molecular events catalyzed by CerS and DES1 corresponding to the gray box on the left are shown in detail, with the catalytic activities of both enzymes highlighted in the gray dotted box areas. **B** Participants exhibit a profoundly high plasma dihydroceramide to ceramide ratio. Results are shown as fold elevation over mean control ratios, which are arbitrarily set at a value of 1. Participant two, black bars; Participant one, gray bars

Because the DES1 protein encoded by *DEGS1* catalyzes the insertion of a double bond into the backbone of sphingolipids during ceramide synthesis (Fig. 5A), we used tandem mass spectrometry to quantify the saturated and unsaturated sphingolipids in plasma samples from participant one and participant two and compared the results to nine pediatric controls. An overall profile of high DHCer levels, low Cer levels, and high DHCer/Cer ratios was evident in both participants compared to controls (Supplemental Table 10; Supplemental Fig. 10). For most lipid species, Cer levels were lower in probands than the average control level. These changes were profound and present across a broad range of DHCer and Cer species, which vary based on the chain length and saturation of the fatty acid component of the sphingolipid. For example, the ratios of C14:0, C16:0, C20:0, C22, C24:0, C24:1 and C26:0 DHCer/Cer were elevated from 42-fold to 44,300-fold in the probands compared to controls (Fig. 5B)., and Cer was significantly lower in participants than controls for C14:0, C16:0, C18:0, C20:0, C22:0, C24:0, C24:1, and C26:0 (Supplemental Fig. 10). These measurements confirmed a functional deficiency of ceramide desaturase activity. Together, these lipidomic analyses demonstrate that this splice site variant of *DEGS1* leads to a loss-of-function of the protein product, DES1.

## Discussion

We identified a new homozygous splice site variant in *DEGS1* c.825+4_825 + 5delAGinsTT, located within the 5’ splice site of *DEGS1* exon 2. The variant was present in three participants with HLD18 from two families of the same ancestry who were unrelated at least to the fourth degree. The available parents were heterozygous for the variant and did not exhibit features of HLD18. The variant was absent from population databases, such as gnomAD, dbSNP, TopMed, DiscovEHR, and had not been previously reported in ClinVar. Through RNA sequencing, cell-based splicing assays, RNA structure probing and mass spectrometry analysis, we conclusively showed that the splice site variant was sufficient to induce exon 2 skipping, and led to the loss of DES1 sphingolipid delta(4)-desaturase protein function. Based on these results, we are submitting the variant to Clinvar as Likely Pathogenic for HLD18.

Interestingly, the findings in participants profiled in this study suggest a more severe disease progression of HLD18 compared to most individuals reported by Pant et al. (2019), in which acquired microcephaly was present in 3/19 (vs. 3/3 in this study), independent sitting attained by 8/19 (vs. 0/3), and contractures present in only 2/19 (vs. 2/3). In spite of the presence of a small amount of normally spliced transcript observed in this study, the participants have reduced desaturase activity, accumulation of dihydroceramides, reduction of ceramides, and severe progression. Future studies may examine a correlation between the variable expressivity of the HLD18 phenotype and the level of residual DEGS1 enzyme activity. For full description of clinical findings for the participants in this study and others with HLD18, see Supplemental Table 5.

Quantification of a diverse range of sphingolipids revealed a clear pattern consistent with and pathognomonic of *DEGS1* loss-of-function, accumulation of substrates and paucity of products (Pant et al. 2019). The combination of extremely reduced Cers and elevated DhCers cannot readily be explained by any other enzymopathy or disease state.

Successful splice-modulating ASOs follow a logic to mask splicing silencers to rescue splicing (Hua et al. 2008; Ottesen 2017). Like our 5′ splice site variant in *DEGS1* exon 2, a 5′ splice site variant in *IKBKAP* exon 20 also causes aberrant splicing. Interestingly, *IKBKAP* exon 20 aberrant splicing can be corrected by splice-modulating ASOs targeting splicing silencer elements (Sinha et al. 2018), motivating us to adapt this ASO discovery strategy to *DEGS1* exon 2. While our ASO walk results informed our identification of regulatory sequences that control *DEGS1* exon 2 splicing, it unfortunately did not identify splice-modulating ASOs that enhanced inclusion of the pathogenic variant. This is because the molecular mechanism by which *DEGS1* c.825+4_825 + 5delAGinsTT affects splicing involves not only potentially reduced binding of U1 snRNP due to + 4 and + 5 changes at the 5’ splice site, but also altered accessibility of regulatory elements other than splice site sequences. Our ASO walk results are also consistent with findings that large internal exons, like *DEGS1* exon 2, contain a high density of enhancers that are important for its splicing fidelity (Bolisetty and Beemon 2012). Taken together with our RNA folding models, the ASO walk data suggested that splicing regulatory sequence may become inaccessible in the context of the *DEGS1* variant. This complicated splicing abnormality highlights the need for exploring more ASO design strategies, including: oligo length optimization, target specificity, and specific chemical modifications (Havens and Hastings 2016).

DNA sequencing-based genetic testing of children with a suspected leukodystrophy has a relatively high yield of specific diagnoses (Zerem et al. 2023). However, this high-throughput testing modality gives rise to numerous VUSs (Kemp et al. 2023; Hamdan and Alasmar 2023). While there are examples of RNA sequencing (Borja et al. 2022), splicing assays (Yan et al. 2021), and metabolic profiling (Pant et al. 2019; Zandl-Lang et al. 2023) being used to establish the pathogenicity of VUSs in individuals with leukodystrophies, these approaches are currently underutilized.

In this study, we utilized a combination of RNA and protein studies to conclusively demonstrate that a 5’ splice site mutation of *DEGS1* exon 2 is pathogenic, highlighting the collaborative interdisciplinary approach that was needed to resolve a VUS and establish the molecular mechanisms of disease. Given the high rate of VUSs, as genomic sequencing becomes more common in clinical practice, we must establish cost-effective and scalable frameworks for their resolution using multi-disciplinary approaches.

## Supplementary Information

Below is the link to the electronic supplementary material.

Supplementary Material 1

Supplementary Material 2

## Data Availability

Sequencing data has been uploaded to the Analysis Visualization and Informatics Lab-(AnVIL) at the National Human Genome Research Institute. Clinical data is available from the authors on reasonable request.
