# Supplementary material for: A novel splice site variant in DEGS1 leads to aberrant splicing and loss of DEGS1 enzyme activity, a VUS resolved
Source: Hum Genet. 2026 May 6;145(1):41. doi: 10.1007/s00439-026-02830-9 (PMC13149559; doi:10.1007/s00439-026-02830-9)
Supplement: Supplementary file 2 — Supplementary Material 2 [file 439_2026_2830_MOESM2_ESM.docx]

## Supplemental Figures with Legends

**
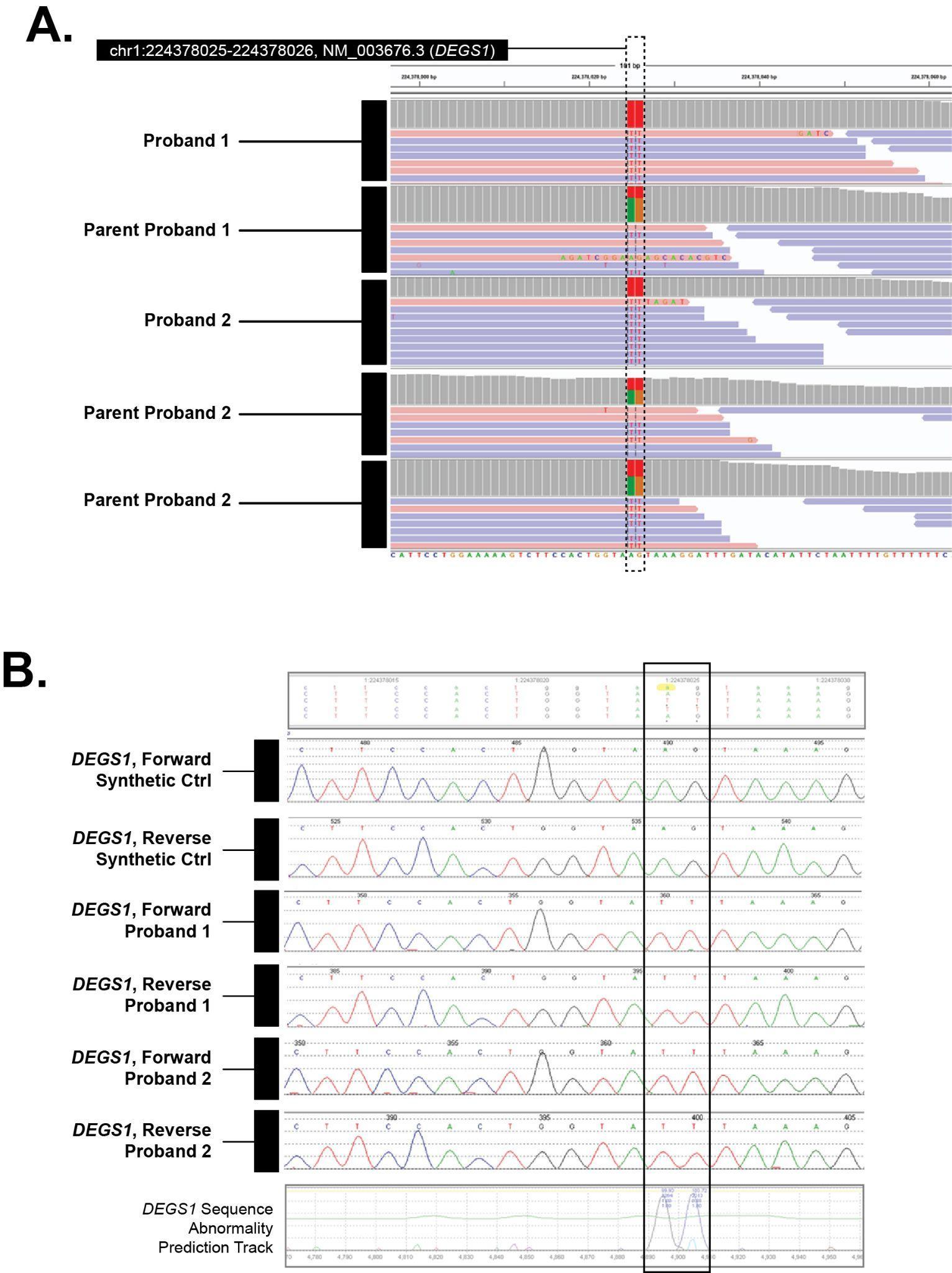
**

**Supplemental Figure 1. Homozygous non-canonical *DEGS1* splice site variant detected in two participants with hypomyelinating leukodystrophy 18.** **(A)** Integrative Gene Viewer plots from exome sequencing showing homozygous variants in two participants and heterozygous variants in available parents. The variants map to the 5' splice site of exon two from the *DEGS1* gene (NM_003676, chromosomal position chr1:224378025-224378026 on hg19). The position of the variant is boxed. **(B)** Sanger sequencing confirming GRCh38NC_000001.11:g.224190323_224190324delinsTT to be homozygous in participants one and two.


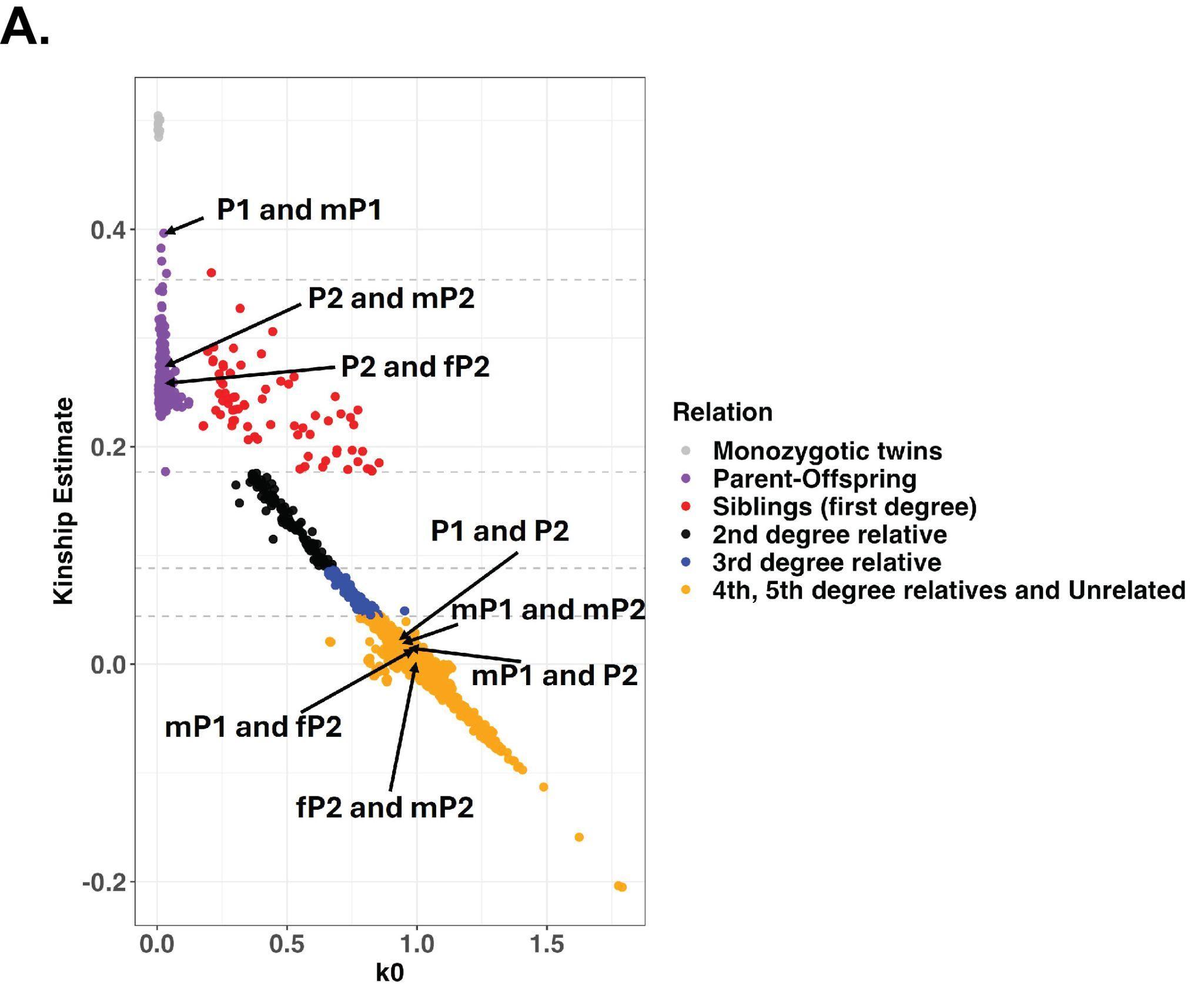


**Supplemental Figure 2. Relationships among participants in the Program in Prenatal and Pediatric Genomics Sequencing (P^3^EGS) study.** Each dot represents a pair of individuals among the sequenced P^3^EGS participants and parents. The Y axis shows the kinship coefficient, which measures how genetically similar two individuals are. Higher values mean the individuals are more closely related. The x-axis (k0) shows the chance that any shared allele between two individuals is identical by state (IBS). IBS means the alleles are the same, whether they came from a common ancestor or not. P1 and P2 represent participants one and two respectively. Female and male parents of the participants are represented by fP and mP, respectively.


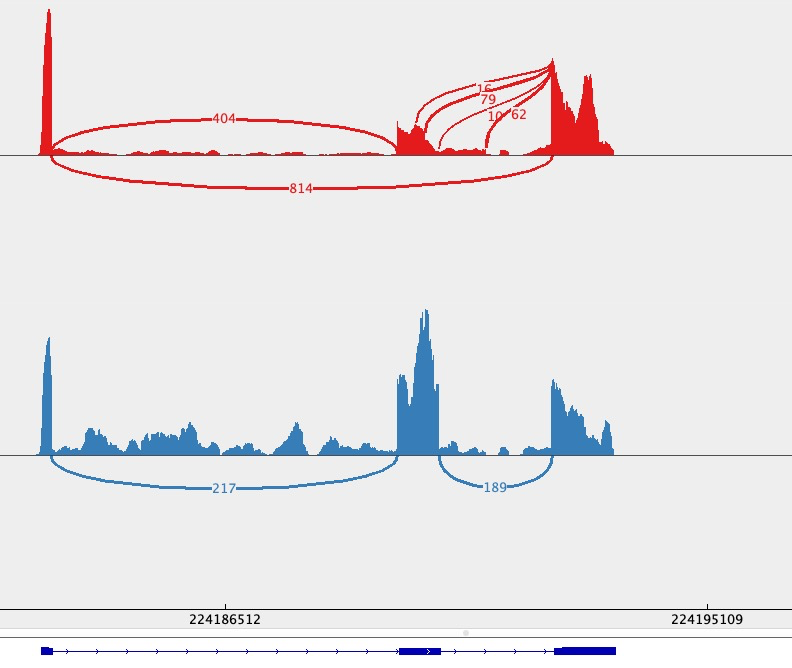


**Supplemental Figure 3. Sashimi plot** **of reads supporting DEGS1 junctions in RNA-Seq data from participant one (red) and control (blue).** Only junctions with 10 or more supporting reads are included. Genomic coordinates are on GRCh38 chromosome one.


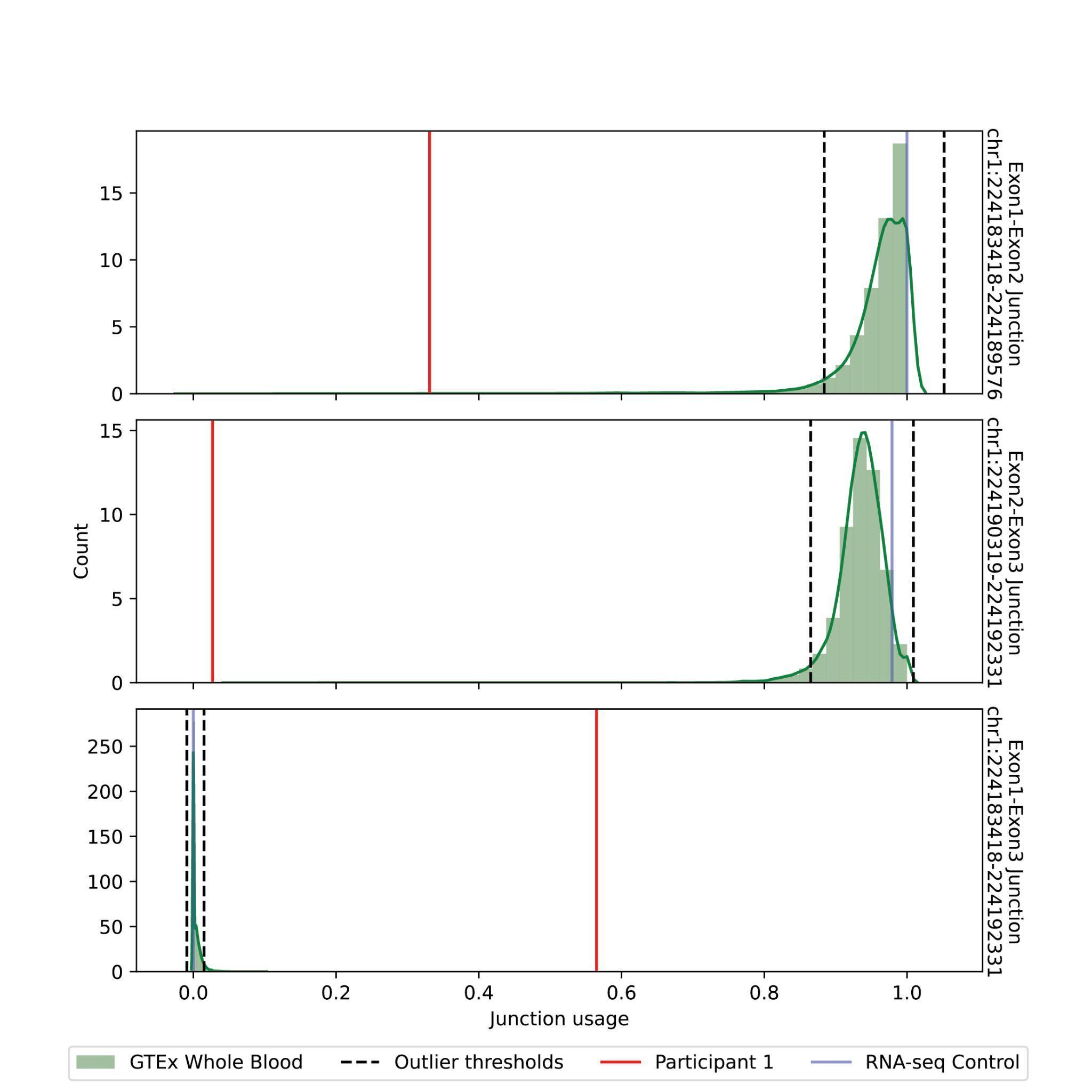


**Supplemental Figure 4. Junction usage in participant one is exceptional relative to the control sample and whole blood in GTEX.** Participant one has exceptionally low usage of commonly used *DEGS1* junctions (top two panels), far below the outlier thresholds defined by junction usage in whole blood in GTEx. Usage of the *DEGS1* exon 1-3 junction is extremely rare in the control sample and GTEx whole blood, but abundant in Participant one. Genome coordinates are for hg38.


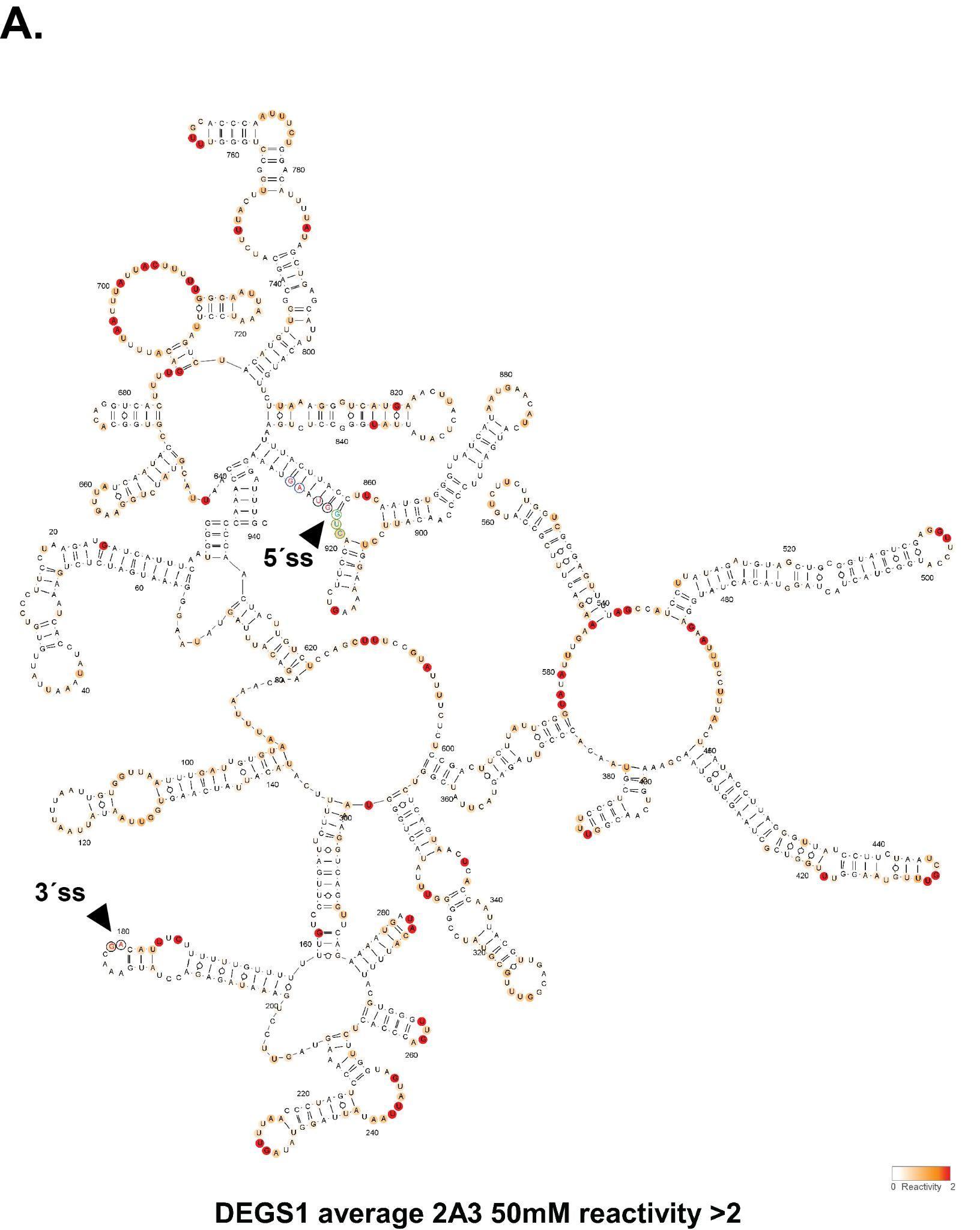


**Supplemental Figure 5.** **Two-dimensional RNA structure prediction for the reference context of *DEGS1* exon two.** SHAPE-derived secondary structural model for *DEGS1* exon two reference and flanking intron sequences, as assayed in splicing reporter assays. Bases are colored according to their normalized 2A3 SHAPE reactivity. All nucleotide position numbering shown is based on the IVT RNA template used for SHAPE probing, from the 5′ to 3′ orientation. Splice sites are indicated with black arrows and appropriate text.


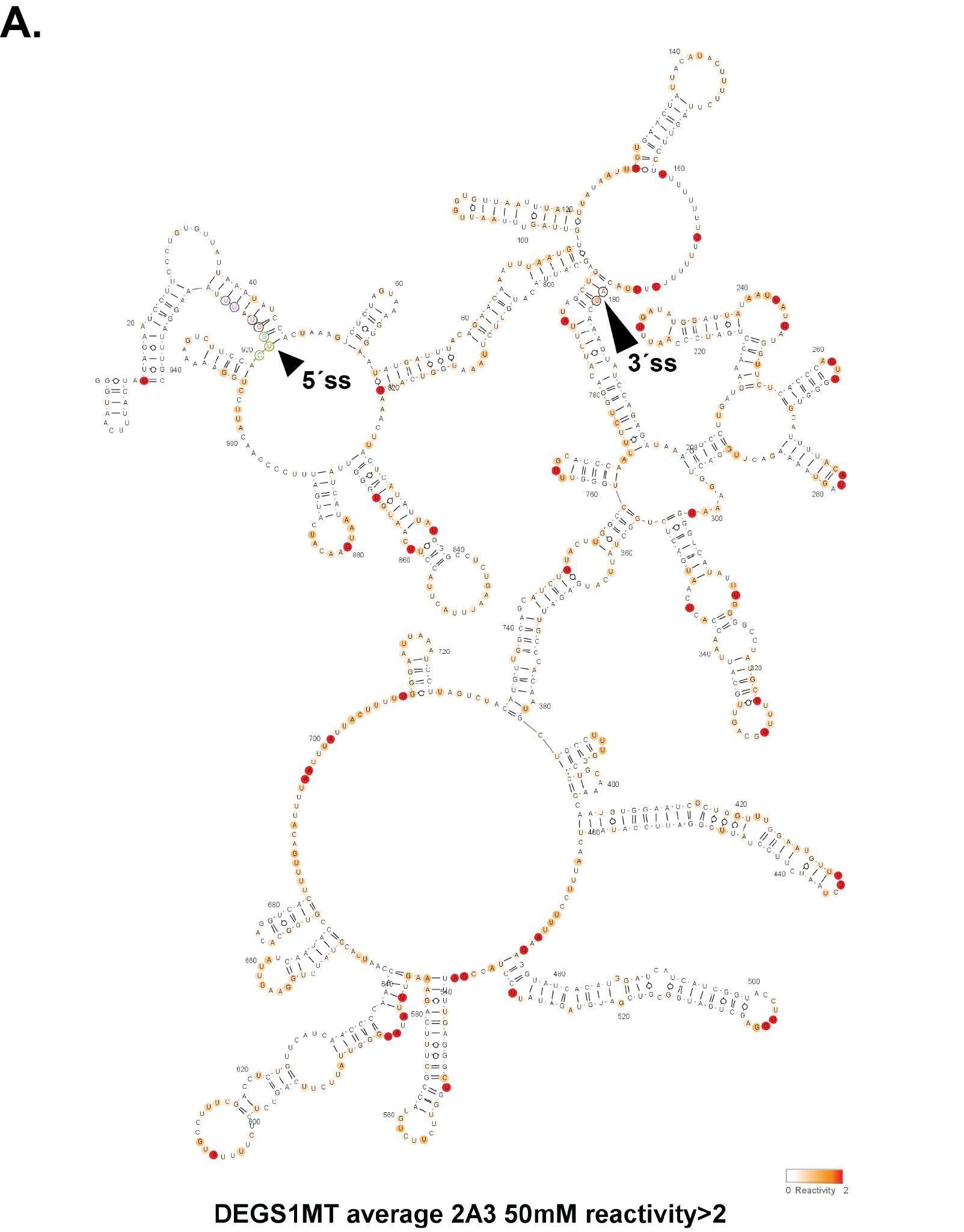


**Supplemental Figure 6.** **Two-dimensional RNA structure prediction for the splice site variant context of *DEGS1* exon two.** SHAPE-derived secondary structural model for *DEGS1* exon two variant and flanking intron sequences, as assayed in splicing reporter assays. Bases are colored according to their normalized 2A3 SHAPE reactivity. All nucleotide position numbering shown is based on the IVT RNA template used for SHAPE probing, from the 5′ to 3′ orientation. Splice sites are indicated with black arrows and appropriate text.


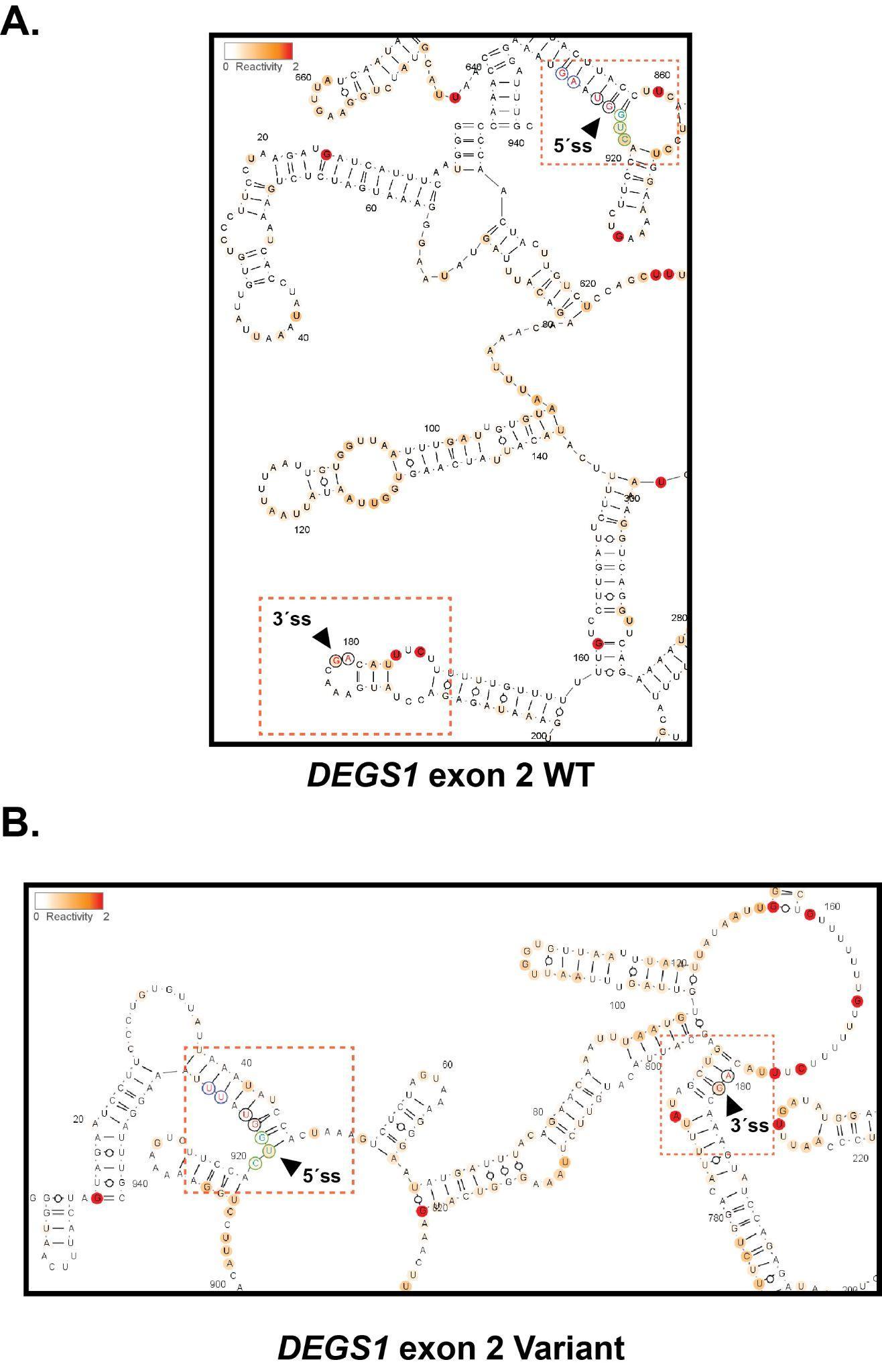


**Supplemental Figure 7. Examining the structural accessibility of splice sites between the reference and variant context of *DEGS1* exon two.** The figure shows the splice sites for the reference (i.e., wildtype; WT), as shown in Panel **(A)**, and for the variant, as shown in Panel **(B)**. Respective splice sites are indicated with a black arrow and are also boxed in red.


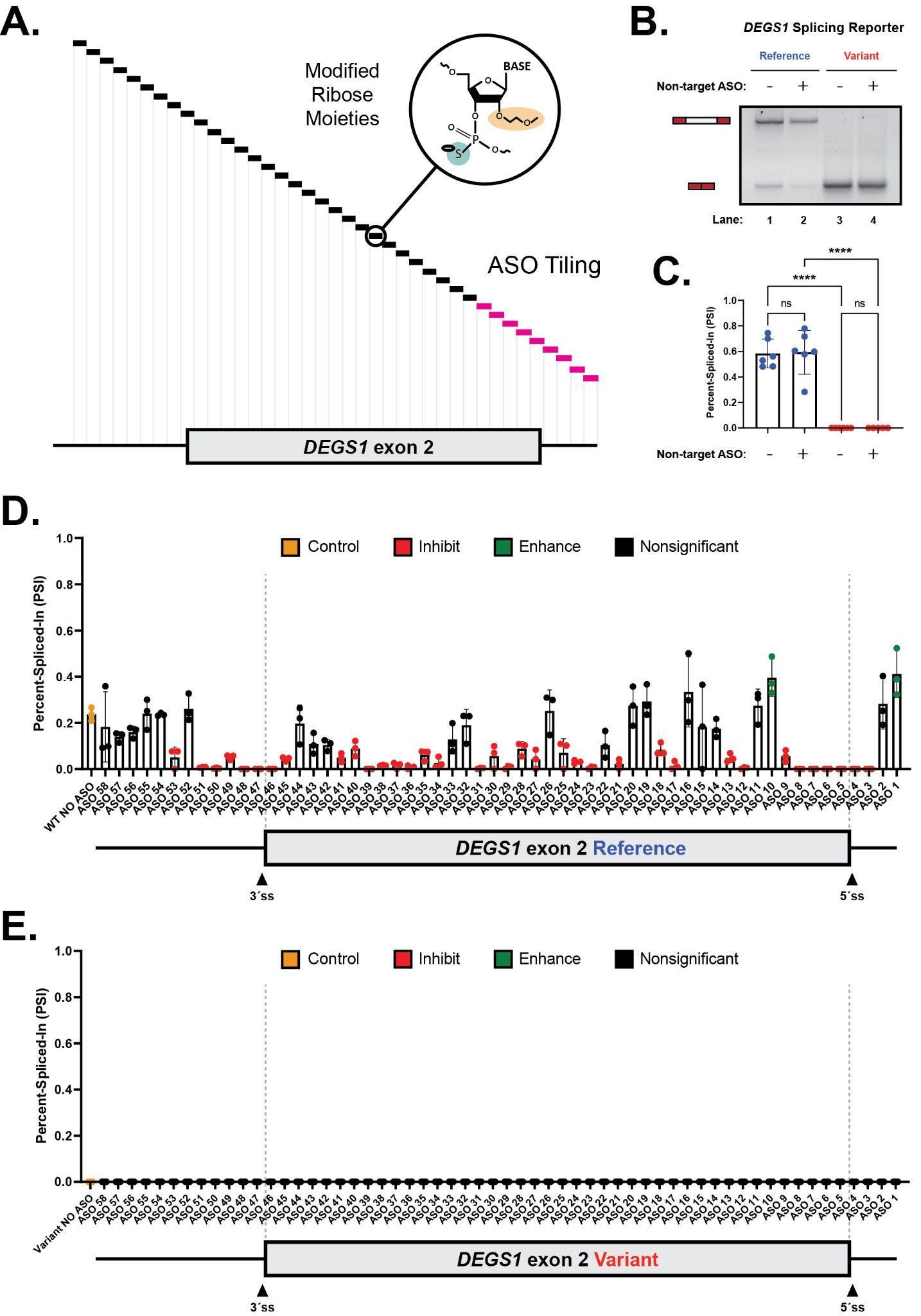


**Supplemental Figure 8.** ***DEGS1* exon two is highly dependent on exonic splicing enhancers for its splicing.** **(A)** A mock schematic of the antisense oligonucleotide (ASO) walk conducted in this study. All ASOs tile across *DEGS1* exon two and its flanking introns. Each ASO used in our walks was 18 nucleotides in length and was designed using ribose sugars that were modified with a 2′-methoxyethyl group (2′-MOE, highlighted in light orange), and the phosphate backbone was modified to a phosphorothioate backbone (highlighted in light blue). Black boxes represent 18-mer ASOs that were contiguous by design, whereas hot-pink boxes represent 18-mer ASOs that had 10 nt overlaps between the preceding and proceeding ASO. **(B)** A representative agarose gel demonstrated no significant difference between conditions with and without a non-targeting ASO being co-transfected with our reference (blue) or variant (red) splicing reporter. Expected mRNA isoforms including or excluding *DEGS1* exon two are also annotated to the left of the agarose gel. **(C)** Percent-Spliced-In (PSI) plot quantifying the non-targeting ASO’s impact on *DEGS1* exon two splicing as shown in (B). **(D, E)** PSI plots quantifying our ASO walk data on the sequence context corresponding to the reference or variant of *DEGS1* exon two, respectively. A schematic model of *DEGS1* and its flanking introns is shown at the bottom of each PSI plot to illustrate relative positions of ASOs, and for which sequence context. ASOs that significantly inhibited splicing are indicated in red, whereas those that significantly enhanced splicing are indicated in green. The control ASO is depicted in yellow, and non-significant ASOs are depicted in black. Statistical significance between comparisons shown is denoted by asterisks (i.e., ****) that represent *P* ≤ 0.0001. Statistical significance was determined using analysis of variance (ANOVA), and Dunett’s post-hoc test. Each condition tested and presented in this figure contains a minimum of three independent/biological replicates.


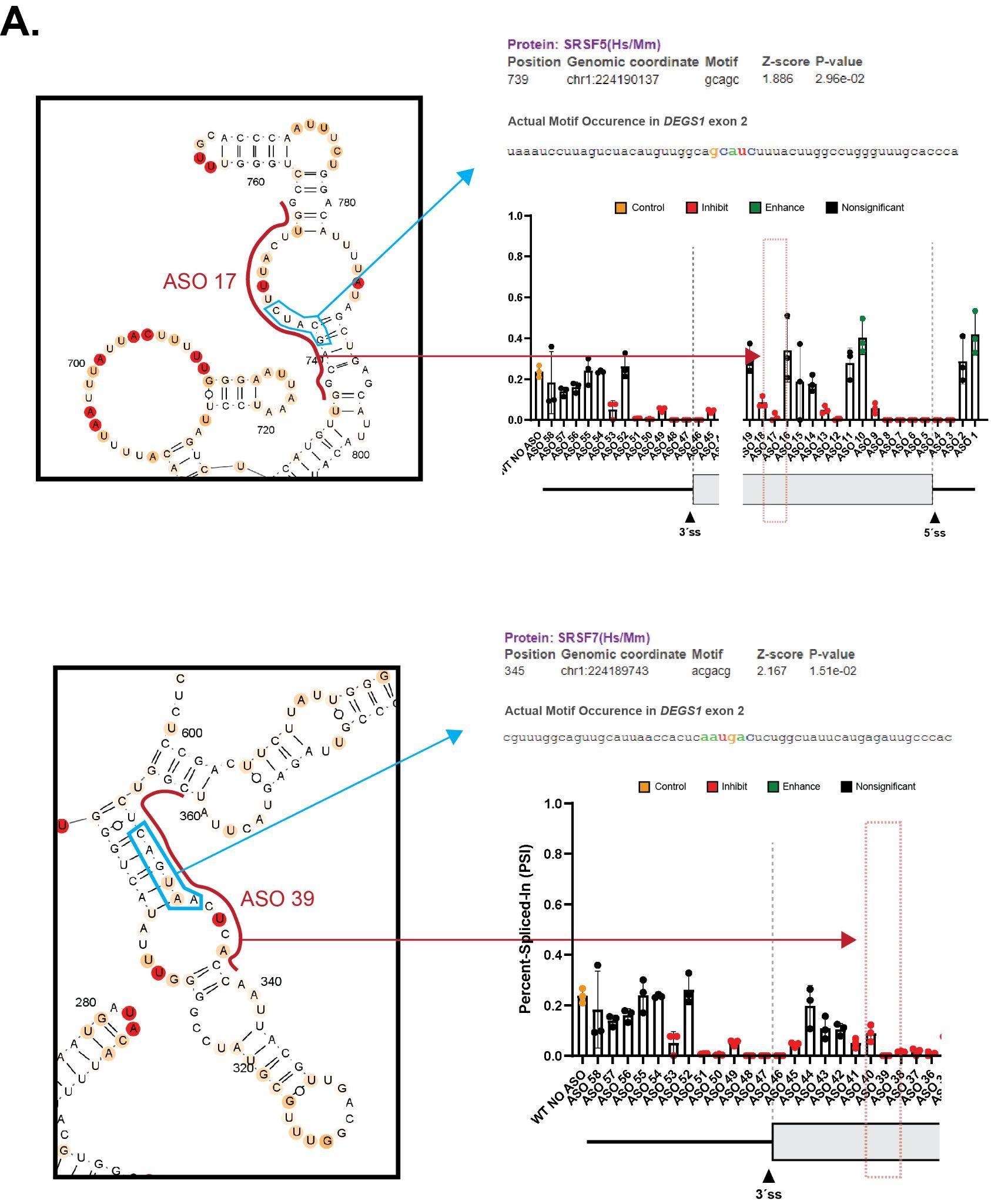


**Supplemental Figure 9. Cross-referencing RBPmap predictions to *DEGS1* exon two SHAPE and ASO data.** Among a list of predictions, for example, RBPmap predicts two putative binding sites for splicing factors known to enhance splicing. The splicing factor implicated is described, showing their motif confidence score and their position within our sequence context assayed, as indicated following the light blue arrow. The effect an ASO has in interfering with these putative splicing enhancers are indicated with red annotations such as text, arrows, and dashed boxes.


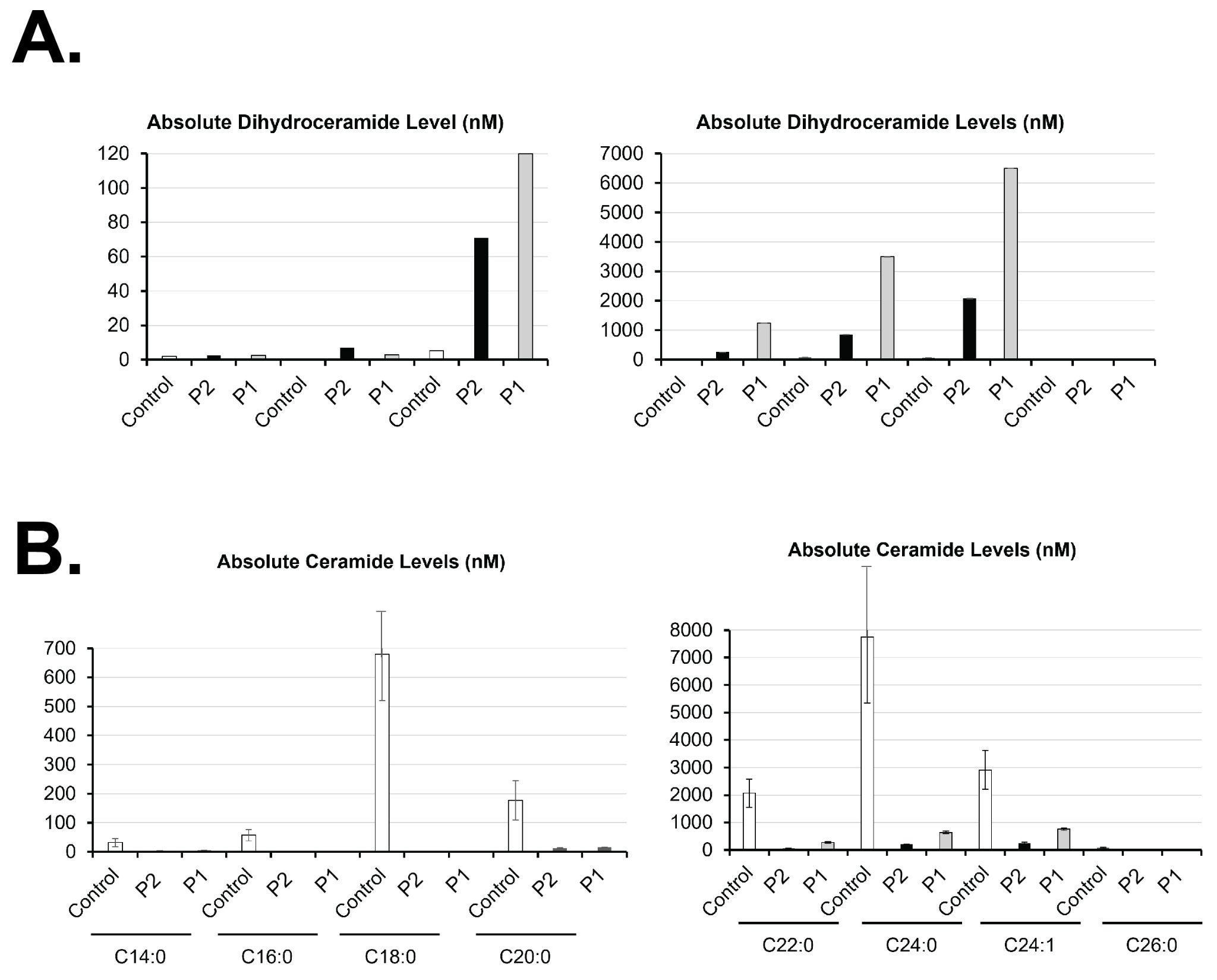


**Supplemental Figure 10. Participants exhibit high levels of dihydroceramides and low levels of ceramides in plasma. (A)** Two patients with homozygous *DEGS1* splice site variants (Participant one (P1) and participant two (P2)) exhibit profound accumulation of the *DEGS1* dihydroceramide substrates in their plasma compared to healthy pediatric controls (n=9). Shown are results for seven dihydroceramide species with fatty acids of the following chain length and saturation (C16:0, C18:0, C20:0, C22:0, C24:0, C24:1, C26:0). **(B)** Two patients with homozygous *DEGS1* variants (P1, P2) exhibit profound deficiency of the *DEGS1* ceramide products in their plasma compared to healthy pediatric controls (n=9). Shown are results for eight ceramide species with fatty acids of the following chain length and saturation (C14:0, C16:0, C18:0, C20:0, C22:0, C24:0, C24:1, C26:0). Results from P2 and P1 are represented by black bars and gray bars, respectively. Results from a control participant are represented by white bars.
